# Fossil ginkgophyte seedlings from the Triassic of France resemble modern *Ginkgo biloba*

**DOI:** 10.1186/1471-2148-13-177

**Published:** 2013-08-27

**Authors:** Kathleen Bauer, Lea Grauvogel-Stamm, Evelyn Kustatscher, Michael Krings

**Affiliations:** 1 Museum of Nature South Tyrol, Bindergasse 1, Bolzano/Bozen 39100, Italy; 2 Department für Geo- und Umweltwissenschaften, Paläontologie und Geobiologie, Ludwig-Maximilians-Universität, Richard-Wagner-Straße 10, Munich 80333, Germany; 3 4 Place du Marché aux Poissons, Strasbourg 67000, France; 4 Bayerische Staatssammlung für Paläontologie und Geologie, Richard-Wagner-Straße 10, Munich 80333, Germany

**Keywords:** Ontogeny, Germination, Cotyledon, Ginkgophyta, Gymnosperms, Middle Triassic

## Abstract

**Background:**

Fossil evidence of ginkgophyte ontogeny is exceedingly rare. Early development in the extant *Ginkgo biloba* is characterized by a series of distinct ontogenetic stages. Fossils providing insights into the early ontogeny of ancient ginkgophytes may be significant in assessing the degree of relatedness between fossil ginkgophytes and *G. biloba*.

**Results:**

An assemblage of seedlings from the early Middle Triassic of France is assigned to the ginkgophytes based on leaf morphology. The specimens represent an ontogenetic sequence consisting of four stages: (I) formation of the cotyledons in the seed and germination; (II) development of primary leaves and taproot; (III) thickening of the taproot and appearance of secondary roots; and (IV) development of the first differentiated leaves and absence of the seed remnants.

**Conclusions:**

The fossil seedlings provide a rare opportunity to examine the early ontogeny of a Triassic ginkgophyte. Germination and seedling development in the fossil are nearly identical to that of the extant gymnosperm *G. biloba*. We hypothesize that the fossil may be closely related biologically to *G. biloba,* and that certain developmental processes in seedling development were in place by the Middle Triassic.

## Background

Fossil evidence of gymnosperm ontogeny is exceedingly rare and mostly limited to isolated specimens of embryos and/or seedlings [1]. For example, structurally preserved araucariaceous embryos *in situ* have been described from the Jurassic of Patagonia [2,3], and impression fossils of araucariaceous seedlings are known from the Jurassic of Brazil [4]. Compressed seedlings attributed to the herbaceous conifer *Aethophyllum stipulare* have also been reported from the Middle Triassic Voltzia Sandstone of France [5], and a conifer embryo with cotyledons has been described from the Upper Pennsylvanian or Lower Permian of North America [6]. Finally, Rydin et al. [7] and Dilcher et al. [8] report on Welwitschiaceae seedlings from the Cretaceous of Brazil.

The ginkgophytes (Ginkgophyta) are a distinctive lineage of gymnosperms that is hypothesized as originating in the late Palaeozoic [9]. The group is considered poorly diversified during the Permian, with a major radiation commencing in the Middle or Late Triassic [10,11]. Today Ginkgophytes are represented by a single species, *Ginkgo biloba*, the maidenhair tree. The early ontogenetic development of *G. biloba* has been studied and documented in great detail [12-16]. Sprecher [13] defines four distinct ontogenetic stages in the early development of *G. biloba*, beginning with germination and ending with the development of the first fully differentiated leaves. To date, the early ontogenetic development of fossil representatives of the ginkgophytes has remained unknown due to a lack of fossils that could be used to reconstruct this process.

The occurrence of well-preserved fossil seedlings in the early Middle Triassic Voltzia Sandstone of the Vosges (northeastern France) has been noted by Grauvogel [17] and Grauvogel-Stamm & Grauvogel [18]. However, none of these fossils have been described in detail and compared to modern equivalents. In this paper, we describe an assemblage of 20 compression specimens of seedlings from a single bedding plane in the Voltzia Sandstone. The seedlings are assignable to the ginkgophytes based on leaf morphological features, and thus represent the first record of seedling development of a fossil ginkgophyte. What makes these fossils even more significant is that the specimens span a number of different ontogenetic stages thus making it possible to compare several stages of seedling development directly with those of the modern taxon.

## Results

The assemblage consists of 20 specimens, 18 of which are seedlings in different stages of development (Table 1).

**Table 1:** Measurements of seeds and seedlings

**Specimen**	**Seed**	**Cotyledons**	**Primary leaves**	**Number of bifurcations**	**Taproot**	**“True” leaves**
Ba25I	8x8 mm	2	not yet	not yet	not yet	not yet
Ba25II	6x5,5 mm	2	not yet	not yet	not yet	not yet
Ba03	not preserved	?2 bases	fragmentary	2	no	not yet
Ba15	not preserved	2 bases	fragmentary	2	not preserved	not yet
Ba17	not preserved	1 base preserved	fragmentary	?	not preserved	not yet
Ba18	not preserved	not preserved	?	?	not preserved	not yet
Ba23	3,4x3,6 mm	2 bases	2 with linear segments	2	no secondary roots	not yet
Ba22	not preserved	2 bases	2 with linear segments	2	with secondary roots	not yet
Ba30	not preserved	not preserved	fragmentary	1(?2)	not preserved	not yet
Ba31	not preserved	not preserved	3	2	no secondary roots	not yet
Ba21I	not preserved	none	2 with linear segments	2	with secondary roots	1
Ba21II	not preserved	none	2 with linear segments	2	fragmentary	fragmentary
Ba21III	not preserved	none	2 with linear segments	2	fragmentary	1
Ba24	not preserved	2 bases	2 with linear segments	2	with secondary roots	not yet
Ba26	not preserved	none	3	2	with secondary roots	1
Ba27	not preserved	2 bases	?	3	not preserved	4
Ba28	not preserved	2 bases	2 with linear segments	2	with secondary roots	not yet
Ba29	not preserved	fragmentary	2 with linear segments	3	with secondary roots	not yet
Ba32	not preserved	fragmentary	2 with linear segments	3	with secondary roots	not yet
Ba33	not preserved	fragmentary	fragmentary	2	with secondary roots	not yet

**Figure 1:**
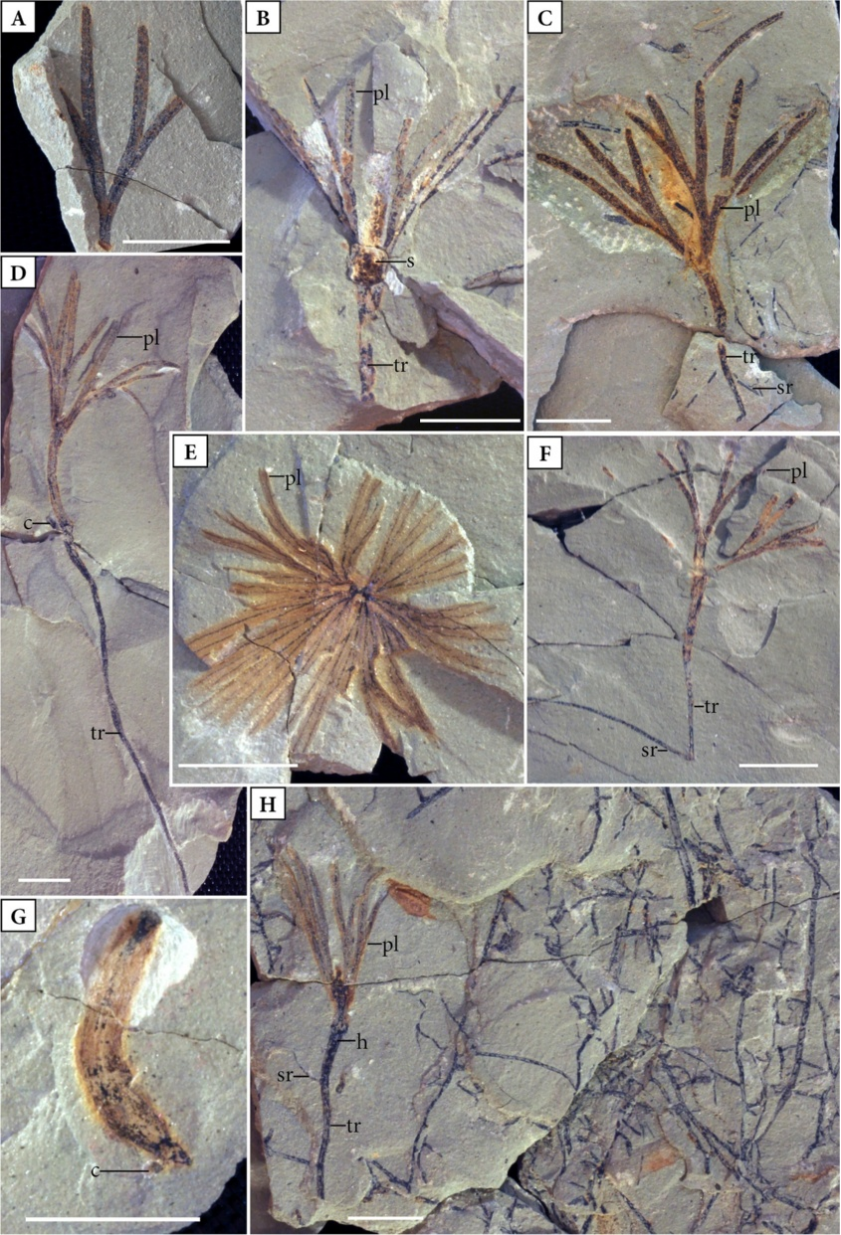
**Scale bar – 1 cm. A** Ginkgophyte leaf, Hangviller (Ba19). **B** Ginkgophyte seedling, stage II,, Adamswiller (Ba23). **C** Ginkgophyte seedling in transition from stage III to IV, taproot broken, Adamswiller (Ba32). **D** Ginkgophyte seedling, transition from stage III to IV, Adamswiller (Ba22). **E** Ginkgophyte seedling, stage IV, top of Figure 1G, Adamswiller (Ba27). **F** Ginkgophyte seedling, transition from stage II to IV, Adamswiller (Ba26). **G** Ginkgophyte seedling, stage IV, Adamswiller (Ba27, back side). **H** Ginkgophyte seedling in transition from stage III to IV, Adamswiller (Ba 24). (c) attachment of cotyledon; (tr) taproot; (sr) secondary root; (s) seed; (h) hypocotyl); (pl) primary leaves.

Five specimens (Ba03, 15; 17; 18; 23**,** Figure 1B) show the earliest stage of development. The seedlings range from 12.8–29 mm long, and are characterized by a small axis, 2.5–17 mm long and 1–4 mm wide. Each axis bears two primary leaves that are twice-bifurcate; leaf segments are linear, 20–22 mm long, and slightly tapered toward the tip. The remnant of the seed, if still present, is located near the axil of the cotyledon and primary leaf (Ba23, Figure 1B).

**Figure 2:**
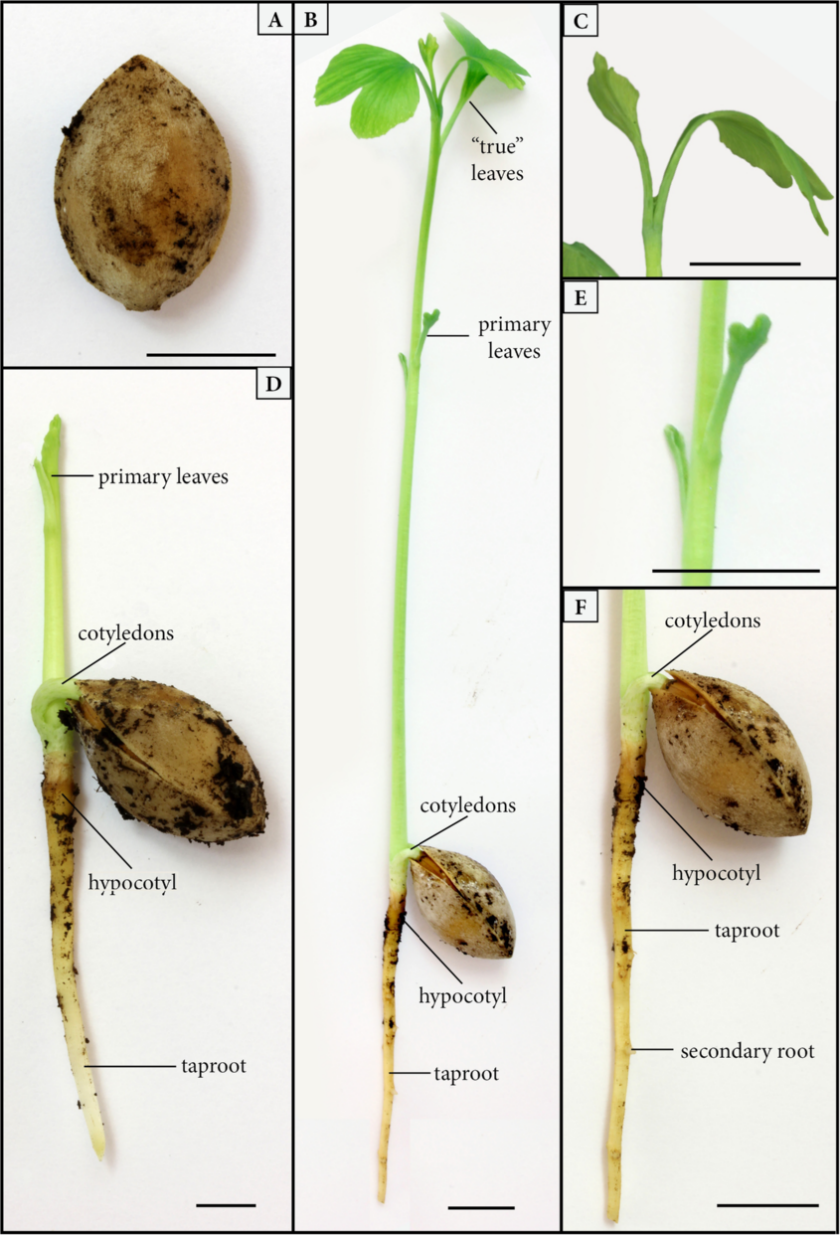
**Scale bar – 1 cm. A ***Ginkgo biloba* seed, stage 0–I. **B***Ginkgo biloba* seedling, stage IV showing taproot, first leaves and differentiated leaves. **C***Ginkgo biloba* seedling, stage IV with oppositely standing leaves. **D***Ginkgo biloba* seedling, stage II with not ramified taproot and first leaves. **E***Ginkgo biloba* seedling, stage IV (primary leaves of (B) enlarged). **F***Ginkgo biloba*, stage IV (enlarged taproot of (B) showing attachment points of secondary roots).

Other specimens demonstrate a slightly more advanced stage of development (Ba22, 30, 31; Figure 1D). Two or three primary leaves are attached to the axis (17–19.8 mm long) that are once- or twice-bifurcate and 17–22 mm long (Ba22, Figure 1D). The linear leaf segments are between 1.35–1.6 mm wide and approximately 10–12 mm long; the tips are acute to slightly rounded (Ba22, Ba30, Figure 1D). What is interpreted as the attachment area of a cotyledon or the seed (Figure 1H) occurs near the transition from the hypocotyl to the epicotyl in the form of a small structure (2 × 3 mm). In some specimens it appears that the hypocotyl region is slightly thickened (Figure 1C, D, H). Whether or not this thickening corresponds to the basal lignotuber formation in *Ginkgo biloba* (Figure 2B, D, F; [14,16]) cannot be determined at this time. The taproot is at least 56 mm long and 1.1 mm wide proximally, but decreases in width towards the tip.

Most of the seedlings (i.e. Ba21I–III; Ba24, 26–29, 32, 33, Figure 1E-H) are 30–50 mm long and possess 2–4 primary leaves, which are bifurcated twice or three times. The taproots are 7.5–39 mm long and approximately 1 mm wide at the hypocotyl. Secondary roots are narrower (Ba24, Figure 1H). The hypocotyl is well differentiated. Tangled masses of ramifying delicate rootlets occur in close association with some of these seedlings, but none appear to be physically connected (Ba24, Figure 1H).

One specimen (Ba27; Figure 1E, G) probably possesses 4 leaves, each of which bifurcates three times. These leaves are linear with the individual segments slightly tapering toward the tips. Part of the shoot axis, as well as the transition from the hypocotyl to the epicotyl, is recognizable within the sediment. In this specimen, the root is not preserved. Adhering to the lower portion of the axis is a small structure that might be the attachment of a cotyledon.

**Figure 3:**
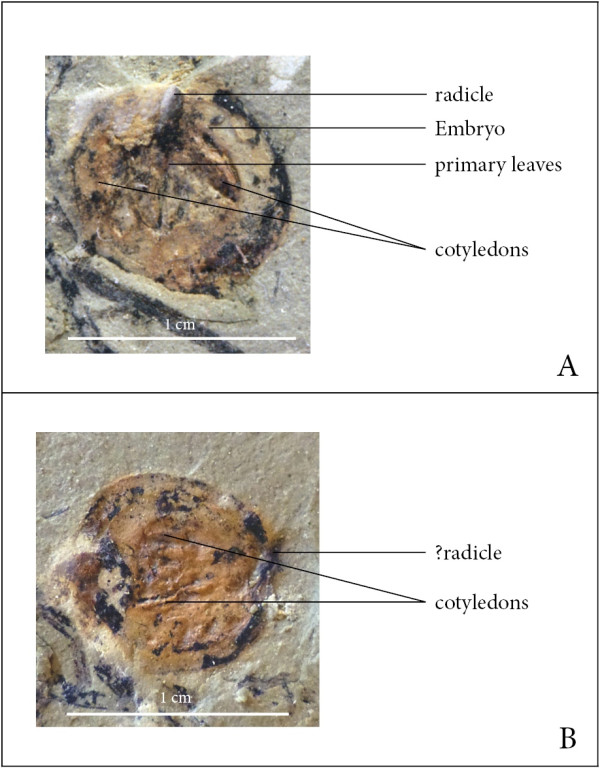
**Scale bar – 1 cm.** Putative ginkgophyte seed (**A**, **B** part and counterpart), showing embryo and early germination (Stage I).

Two slabs contain isolated seeds of uncertain systematic affinities. The seeds are elongate-elliptical and up to 0.9 cm long and 0.8 cm wide (Figure 3A, B). In one of the seeds, outlines of interior structures probably resembling the embryo with cotyledons and vegetative primordial of the primary leaves are faintly visible.

## Discussion

The assemblage of Middle Triassic seedlings from the Voltzia Sandstone of France provides a remarkable sequence of stages in seedling morphology of a Mesozoic gymnosperm. Of particular interest is the fact that the specimens are not all preserved at the same stage of development, but rather represent a variety of ontogenetic stages which can be used to trace the sequence of morphological patterns for a single taxon.

### Affinities

To date six different plant groups have been reported from the Voltzia Sandstone, i.e. lycophytes, sphenophytes, ferns, conifers, cycadophytes, and ginkgophytes. Lycophytes, sphenophytes, and ferns can be ruled out as the producers of the seedlings based on their life history biology. Cycads can also be eliminated because the cotyledons of cycad seedlings remain within the seed coat until well after the root system is fully established in the soil [19]. Moreover, upon emergence, the cotyledons of cycads often remain intimately fused for some time. In addition, the leaves of older cycadophyte seedlings are pinnate [20]. On the other hand, the primary leaves of conifers occur singly and are not bifurcated [5]. Finally, the epigeal seedlings of conifers typically possess more than two cotyledons [4,21], while the fossil seedlings reported here (where preserved) have two cotyledons.

Based on this analysis the seedlings from the Voltzia Sandstone were most likely produced by a ginkgophyte. Their precise affinity within the ginkgophytes remains unresolved, however, we hypothesise that the morphology of the differentiated leaves in some of the fossils (e.g., Ba22, 23, 32, Figure 1B-D), together with the linear shape of the leaf segments, strongly suggests affinities with the genus *Baiera*. Interestingly *Baiera* leaves have been described previously from the Voltzia Sandstone (e.g., [5], pl.5, 3; [22]). Moreover, the overall similarity of the primary leaves, together with the close spatial co-occurrence of the fossils, suggests that all seedlings belong to the same species, with the possible exception of Ba27, which is characterized by four leaves that are three-times bifurcated, whereas all other seedlings have two or three leaves, each of which twice bifurcate.

The systematic affinities of the seeds (Figure 3) co-occurring with the seedlings in the Voltzia Sandstone cannot be determined. However, it is likely that they also belong to the ginkgophytes based on similarities in size and overall morphology to seeds assigned to this group of plants from elsewhere (e.g., [23-26]).

### Taphonomy

Sediment structure and fossil content of the Voltzia Sandstone are suggestive of a deltaic sedimentary environment and semi-arid, seasonal climate [27,28]. Thick lenses of fine-grained sandstone correspond to point bars deposited in sinuous channels, whereas silt-clay lenses represent deposits of finer material in brackish ponds scattered between the channels. The fact that most of the seedlings are preserved intact suggests that they were not submerged in water over extended periods of time and were probably not transported over long distances after having been washed out from their place(s) of growth. Undoubtedly, a fortunate (and thus very rare) combination of depositional environment, germination time, and ecological conditions has resulted in the quality of preservation displayed by these fossils. The seedlings are embedded in fine silt-clay sediment, which probably represents a deposit formed in the small ponds and pools. We are uncertain whether this indicates that the seedlings grew along the margins of these bodies of water, or grew at some distance and became washed into the ponds during a flooding event. However, the fidelity of preservation of delicate structures such as seedlings would appear to argue in favour of the former.

### Ontogenetic development of the fossil ginkgophytes

The seedlings from the Voltzia Sandstone reflect three subsequent stages in the early ontogenetic development of a Mesozoic ginkgophyte. While pre-germination conditions and germination (stage I) cannot be unequivocally documented for this ginkgophyte based on the fossils at hand (see below), the second stage (stage II) is characterized by the development of a short shoot axis, a typical gymnosperm taproot, and the appearance of the first 2–4 leaves (the primary leaves). These leaves already possess the typical ginkgophyte bifurcation and are once to three-times subdivided into linear segments. Remnants of the seed are still attached to the seedling. During the subsequent stage III, the taproot thickens and produces delicate lateral (secondary) rootlets (Figure 1H). In stage IV, the shoot axis (or epicotyl, see Taylor et al. [9]: 1037) continues to elongate. Moreover, toward the end of the early ontogenetic development, the first “true” leaves emerge (Figure 1E). Like the primary leaves, these leaves are fully differentiated and characterized by multiple linear segments (Figure 1E). While the attachment points of the cotyledons are still recognizable on the shoot axis in the oldest seedlings (stage IV) from the assemblage, remnants of the seed coat are absent (Figure 1G). At the end of the sequence, the juvenile plant is established.

If the isolated seeds found in close association with the seedlings (Figure 3A, B) in fact belong to the same plant species as the seedlings, then they would indicate that the earliest phase of the ontogenetic sequence (stage I) in this plant is characterized by the seed containing the embryo with two cotyledons and vegetative primordial of the primary foliage. Moreover, germination (i.e. emergence of the radicle and shoot axis from the seed) marks the end of stage I.

The sequence of early developmental stages depicted above based on fossil seedlings from the Voltzia sandstone closely resemble the ontogenetic stages seen in *Ginkgo biloba* (Figure 2A-F: stage I: 2A; stage II: 2D, stage IV: 2B-C, 2E-F), with the exception that the primary leaves in *G. biloba* are smaller and have a more simple morphology. Moreover, the primary leaves in *G. biloba* lack the typical bifurcation evident in the primary leaves of Mesozoic ginkgophytes. All other aspects of the early developmental stages of the fossil are very similar, if not identical, to that observed in *G. biloba*. Sprecher [13] defines the developmental stages in *G. biloba* as follows: During stage I (germination), the radicle and axis emerge from the seed. Subsequently, the shoot axis elongates, the first leaves (primary foliage) appear, and a taproot is developed (stage II). Secondary roots begin to appear during stage III. Remnants of the seed remain attached to the seedling during almost the entire early development, and first disappear following the maturation of the first fully differentiated (“true”) leaves (stage IV).

## Conclusions

The Triassic ginkgophyte seedlings reported here provide a new complement of morphological and developmental data to our understanding of the paleobiology of the ginkgophytes. The sequence of events representing the early ontogenetic development of plants is governed by a complex series of biochemical and physiological interactions that are in turn directed by the interplay between the genetics of the organism and multiple factors operating within the ecosystem. The discovery of these seedlings provides a rare view of developmental stages of a fossil organism that can be directly compared with those of a well understood living plant. Not only do these fossils indicate that morphological information about seedling developmental stages are preserved, but this discovery also offers another perspective that can be used to indicate similarity between the fossil and extant relative. Although the precise systematic affinities of the ginkgophyte seedlings from the Voltzia sandstone remain unresolved, we hope that additional specimens, including more advanced juvenile plants, will be discovered. Information about seedling morphology provides a new data set that will help contribute to a more robust discussion of not only seed plant phylogeny, but also to our understanding about developmental processes that occurred in fossil plants after embryo formation and during seedling development.

## Methods

### Stratigraphical setting and age

The Voltzia Sandstone (Grès à Voltzia) belongs to the Upper Buntsandstein (Bithynian, early Anisian) [29,30]. The sandstone was deposited in a deltaic environment on the western margin of the German Basin. The succession is ~20 m thick and consists of the Grès à meules and the Grès argileux, which constitutes the transition to the marine Muschelkalk [31].

The assemblage of ginkgophyte seedlings has been collected from a single bedding plane within the Grès à meules (lower Voltzia Sandstone), in which three different facies have been recognized [27,28,32]: (a) thick lenses of fine-grained sandstone, grey or pink but more often multi-colored, containing plant debris and stegocephalian bone fragments; (b) greenish or reddish silt-clay lenses, generally consisting of a succession of thin laminae (each a few mm thick) with well-preserved fossils of aquatic and terrestrial organisms, as well as plant remains; and (c): beds of calcareous sandstone with a sparse marine fauna. The sediments crop out in several quarries in northeastern France that have been actively visited by Louis Grauvogel between 1936 and 1980. Ginkgophyte fossils, including isolated leaves, thus far have been discovered from the silt-clay lenses of facies b in the quarries of Adamswiller, Bust, Gottenhouse, Soultz-les-Bains, Hangviller and Arzviller. The seedlings described in this report come from a single, 60 cm thick, silt-clay lens in Adamswiller (Bas-Rhin, France); all have been collected from the same thin bedding planes. For detailed information on the Adamswiller silt-clay lens, including biota, refer to Gall ([27]; *Baiera* in Figure twenty eight) and Selden and Nudds ([33]; Figure one hundred twenty) in which this taxon is merged in “plant debris”.

For comparison with the fossils, nine seeds of *Ginkgo biloba* were potted and cultivated in a greenhouse over a period of 3 months. Germination and early seedling development were closely monitored and documented photographically (Figure 2A-F).

### Storage and documentation

The material is deposited in the collection of Louis Grauvogel in Ringendorf (France); specimens are preceded by “Ba”. Digital images were captured with a Canon Eos D550 digital camera.

## Competing interests

The authors declare that they have no competing interests.

## Authors’ contributions

LG-S made fossil specimens available; KB, EK, and MK designed research; KB and EK performed research; KB, LG-S, EK, and MK analyzed data and wrote the paper. All authors read and approved the final manuscript.
